# Supplementary material for: Four New Species of Haplocauda, with Notes on the Evolutionary Convergence of Copulation Clamps in Lucidotini (Coleoptera: Lampyridae: Lampyrinae)
Source: Insects. 2025 Aug 8;16(8):824. doi: 10.3390/insects16080824 (PMC12386329; doi:10.3390/insects16080824)
Supplement: Supplementary file 1 [file insects-16-00824-s001.zip › Supplementary_material_S1.pdf]

Supplementary Material S1. Material examined of the outgroup taxa.

Lampyrinae

Lampyrini

Lampyris noctiluca. Italy. Sorrento, 1/VII/1987, J.J.Anderson and Emily Foust col (USNM).

Lucidotini

Alychnus suturalis. (♂, IAvH-E-219233): "COLOMBIA, Cundinamarca, Guasca, Vereda Rincón del Oso, Finca Suasié, 4°43′15.2″ N 73°51′50.6″ W, 3310 m, 15 November 2014, Colecta directa en Páramo bajo, D. Martínez col.//Alychnus suturalis"

Costalampys delicata. Holotype. BRAZIL. Rio de Janeiro: 1♂, Teresópolis, P.N. Serra dos Órgãos, Malaise trap, PVE9B (22°26'57.8"S, 43°0'13.7"W, 1,236 m), I.2016, L. Silveira col. (DZRJ).

Costalampys klugii. Holotype: BRAZIL: 1♂, Without other data (ZIN).

Costalampys tricolor. BRAZIL: Minas Gerais: 1♀, Bocaina de Minas, Ribeirão Santa Casa, prox. Cachoeira da Raposa, 22°18'24.7"S, 44°33'54"W, 1294 m, M&M col. (DZRJ).

Dilychnia guttula. BRAZIL. Acre: Cruzeiro do Sul, Ilha Florianópolis, 1 male, 12–17.V.1981, Norman & Elias (INPA). Brazil. Amazonas. São Paulo Olivença, 10.VIII.1925, 1 female and 1 male, H.L. Boy (MNRJ).

Dadophora hyalina. Brazil. Without other provenance data. 1 Male. (BMNH);

Ethra marginata. Brazil. Rio de Janeiro. Teresópolis, without date, 1 male (DZRJ).

Haplocauda albertinoi. BRAZIL.Acre: Bujari, FES Antimary, 09 20001" S–68 19017" W, 21.x-04. xi, 2016, Malaise grande, E. F. Morato, & J. A. Rafael cols–Rede BIA //HAPLOCAUDA ALBERTINOI HOLOTYPE".

Lucidota atra. United States of America. NC, Cullowhee, VIII/2020, 1 male (WCCA).

Lucidota banoni. French Guiana. Without other provenance data, 1 male, 1 female (DZRJ).

Luciuranus josephi. PARATYPES. BRAZIL. Rio de Janeiro. Teresópolis. PREPVE Pt.5 A 22°27'16.7'' S 43°01'13.7'' W 1961m, 11M, 7F, VIII/2014, Silveira & Khattar col. (DZRJ); idem, 1M, 1F, IX/2014, Silveira & Khattar col. (DZRJ); PREPVE Pt.5 B 22°27'18.0'' S 43°01'12.2'' W 1949m, 1M, 3F, VIII/2014, Silveira & Khattar col. (DZRJ); PREPVE Pt.6C 22°27'18.0'' S 43°01'12.2'' W 2190 m, 2M, 1F, VII/2014, Silveira & Khattar col. (DZRJ); idem, 1F VIII/2014, Silveira & Khattar col. (DZRJ); idem PENSA RIO Pt.5 22°27'18.7'' S 43°01'32'' W 1630m, VIII/2013, 17M, 2F R. Monteiro col. (MZSP); idem PENSA RIO Pt.3 22°26'55.6'' S 43°00'44.3'' W 1250m, 5M, VIII/2013, R. Monteiro col (MNRJ).

Luciuranus sinistrus. PARATYPES. BRAZIL. Rio de Janeiro. Itatiaia. PENSA RIO Pt.1 22°26'01.4'', S 44°36'49.3'' W 1070m, 10M, 15F, X/2014, R. Monteiro col. (DZRJ); PENSA RIO Pt.1 22°26'01.4'', S 44°36'49.3'' W 1070 m, Male, X/2014, R.

Scissicauda disjuncta. Brazil. Rio de Janeiro. Teresópolis, Serra dos Órgãos N. P., 1050m, XII/2013, Malaise trap, 1 male and 1 female, R. Monteiro col. (DZRJ).

Scissicauda antennata. HOLOTYPE (INPA, pinned). BRAZIL, Rondônia: Nova, Mamoré, Parque Estadual de, Guajará-Mirim. Rio Formoso //101926S– 643388W, 20-, 27.x.1995, J. Vidal & L. S., Aquino. Arm. De Malaise// Scissicauda antennata HOLOTYPE [red label].

Scissicauda jamari. HOLOTYPE (INPA, pinned). BRAZIL, RO, Itapuã do Oeste, Flona Jamari, Igarapé Preto, 091116.0" S–625657.0" W//07.x.2014, Varredura, J.A., Rafael, F.F. Xavier F , R.M., Vieira & R.H. Aquino.//Scissicauda jamari HOLOTYPE [red label.

Scissicauda malleri. HOLOTYPE (MNHN). voi Lucidota imcopta. Lucidota malleri. Ma 248, h, 12. TYPE.

Scissicauda biflabellata. HOLOTYPE: (DZUP). Dept Zool, UF-Parana// Brazil. Espirito Santo. Conceição da Barra, 11.9.1969, C & C.T. Elias col.//DZUP 429998// Scissicauda biflabellata, HOLOTYPE, det. Roza 2023."

Scissicauda balena. Holotype, male. Brazil: Espírito Santo, [n] 6521, Descourtils [leg.], coll. Fry 1905-100 (BMNH).

Uanauna angaporan. PARATYPES. Brazil. Rio de Janeiro, Itaguaí, Parque Estadual Cunhambebe, 22º 50' 53.4" S 43º 54' 29.3" W, 150m, IX.2011, 1 male L. Silveira, Clarkson Sampaio & Ferreira-Jr col. (DZRJ); Angra dos Reis, Parque Estadual da Ilha Grande (PEIG), approx. 23º 10' 30.4'' S 44º 11' 11.3'' W, IX.2008, 2 females, Projeto Coleoptera [J. Mermudes] col. (MNRJ).

Ybytyramoan monteirorum. PARATYPE. Brazil. Teresópolis, P.N. Serra dos Órgãos, Represa do Rio Beija-Flor, 980m, 28/XI/ 2013, L. Silveira, R. Monteiro, M. Macedo col. (DZRJ).

Photinus corruscus. UNITED STATES OF AMERICA. North Carolina: Jackson county, Sylva (35.3281198,-83.1756354), VII/2020, 5M, 1F, L. Silveira col. (WCCA)

Photinus pyralis. UNITED STATES OF AMERICA. North Carolina: Jackson county, Sylva (35.3281198,-83.1756354), VII/2020, 2M, 2F, L. Silveira col. (WCCA)

Phosphoanopterus metzneri. Examined from Nunes et al. (2021): Nunes, V., Figueira, G., Lopes, L. F., & Souto, P. (2021, September). On the Natural History of the Black Winged Firefly, Phosphaenopterus metzneri Schaufuss, 1870 with Comparative Notes on Phosphaenina (Coleoptera: Lampyridae). In Annales Zoologici (Vol. 71, No. 3, pp. 661-691). Museum and Institute of Zoology, Polish Academy of Sciences.

Phosphaenus hemipterus. PORTUGAL. Leiria: São de Alge (40°01'22.3''N, 8°15'47.2''W), 1M collected by colored pan traps, 23/V/2019; Coimbra: Fajão (40°09' 54.9''N, 7°55'22.2''W), 1M collected by pitfall traps, 19/VI/2019; Seia: Cabeça (Parque Natural da

Serra da Estrela), 1M collected by pitfall traps (hosted at Museu de História Natural e da Ciência, University of Porto), 16 to 28-VI-1999; Belgium: Herne, 1M collected by active search, 06/VI/2003, Raphaël De Cock col.

Pseudolychnuris vittata. COLOMBIA, Cundinamarca, PNN Chingaza, Alto de la Bandera, 4°31′ N 73°45′ W 3660 m, Malaise 8–22 December 2000, E. Niño leg. M1033//Bradley Smith Proj. '07 079001//Pseudolychnuris L. SILVEIRA det. 2019. (♂, IAvH-078005).

Pyractonema compressicornis. CHILE: Maule Prov., Paso Garcia, 300 m, ca. 23km NW Cauquenes, 29-30 Nov. 1981. D.R. Davis, 1M (USNM)

Pyractonema haemorrhoa. CHILE: Region IX Nahuelbuta NP, S 37.809°, W 73.016°, 3680', 6-9.i.2000, D.W. Webb, D.K. Yeates col., 2M (WCCA)

Pyropyga minuta. UNITED STATES OF AMERICA. NC: Sylva, 09.IX.2020, L. Silveira col. N 35°22'25.35", W 83°15'33.52", 2M (WCCA)

Pyropyga nigricans. UNITED STATES OF AMERICA. Green Valley, Pima Co., Arizona // Dr. Lenczy, 6.1969, 1M (USNM).
